# Reply to Mahida et al.

**DOI:** 10.1093/icvts/ivag119

**Published:** 2026-04-21

**Authors:** Takuya Tokunaga, Go Kamimura, Masaya Aoki, Kazuhiro Ueda

**Affiliations:** Department of General Thoracic Surgery, Surgery and Science, Graduate School of Medical and Dental Sciences, Kagoshima University, Kagoshima City 890-8530, Japan; Department of General Thoracic Surgery, Surgery and Science, Graduate School of Medical and Dental Sciences, Kagoshima University, Kagoshima City 890-8530, Japan; Department of General Thoracic Surgery, Surgery and Science, Graduate School of Medical and Dental Sciences, Kagoshima University, Kagoshima City 890-8530, Japan; Department of General Thoracic Surgery, Surgery and Science, Graduate School of Medical and Dental Sciences, Kagoshima University, Kagoshima City 890-8530, Japan

We thank Mahida et al. for their thoughtful and constructive Letter to the Editor^1^ regarding our recent publication^2^ and their insightful comments on the mechanical interpretation and clinical applicability of stapler-induced parenchymal injury during deep wedge resection.

First, regarding the concern that resection depth alone may be an oversimplified predictor of empty space formation, we agree that it should not be interpreted as an absolute or deterministic parameter. We consider resection depth to be one of several clinically accessible indicators reflecting the extent of lung parenchyma subjected to stapler compression, rather than a definitive surrogate for mechanical failure. Given the limited number of evaluable cases in this exploratory study, our analysis relied on a restricted set of measurable variables. Accordingly, the proposed depth threshold should be regarded as context-dependent, not universal.

Second, we agree that the number of stapler cartridges used is subject to multiple confounding factors. In clinical practice, cartridge usage is influenced by lesion location and depth, parenchymal thickness, and background lung characteristics such as tissue stiffness. Cartridge count was therefore presented not as a mechanistic determinant but as a contextual variable associated with resection complexity. Clinically, increased cartridge usage is often encountered in cases involving deep-seated lesions or relatively firm lung parenchyma, which may predispose tissue to excessive compression. Further case-specific analyses and experimental studies will help clarify the role of stapling conditions in parenchymal rupture.

Third, we concur that porcine lungs cannot be directly equated with human lungs, and that caution is required when extrapolating these findings to clinical practice. In the present study,^2^ porcine lungs were used as an experimental platform to elucidate the mechanical mechanism of compression-induced parenchymal injury. While we cannot conclude that the same process occurs identically in human lungs, our experiments demonstrated a biomechanically coherent mechanism by which excessive compression may result in internal parenchymal rupture without pleural disruption. The animal model should therefore be interpreted as providing mechanistic insight rather than clinical equivalence.

Finally, we agree that the present study does not establish a direct association between compression-induced empty space formation and clinical outcomes, as this investigation was not designed as an outcome-based study. Nevertheless, margin recurrence following wedge resection is a recognized clinical issue, and it is conceivable that the mechanism demonstrated in our study may contribute to such events in selected cases.

We also appreciate the authors’ suggestion that future investigations should integrate patient-specific tissue characteristics, intraoperative mechanical parameters, and long-term oncologic outcomes. From a clinical standpoint, quantitative assessment of lung tissue along the planned resection line using preoperative CT imaging may help estimate whether safe stapling is feasible. In addition, development of an intraoperative device capable of assessing tissue thickness and elasticity at the intended resection line may provide real-time biomechanical information relevant to stapling safety. Accurate evaluation of the true surgical margin, considering the possibility of parenchymal rupture and internal cavity formation, may help determine whether compression-induced empty space formation should influence procedural selection.
